# The potential distribution of cassava mealybug (*Phenacoccus manihoti*), a threat to food security for the poor

**DOI:** 10.1371/journal.pone.0173265

**Published:** 2017-03-15

**Authors:** Tania Yonow, Darren J. Kriticos, Noboru Ota

**Affiliations:** 1 HarvestChoice, InSTePP, University of Minnesota, St. Paul, MN, United States of America; 2 CSIRO, Canberra ACT, Australia; 3 The University of Queensland, School of Biological Sciences, St. Lucia, QLD, Australia; 4 CSIRO, Wembley WA, Australia; University of Thessaly School of Agricultural Sciences, GREECE

## Abstract

The cassava mealybug is a clear and present threat to the food security and livelihoods of some of the world's most impoverished citizens. Niche models, such as CLIMEX, are useful tools to indicate where and when such threats may extend, and can assist with planning for biosecurity and the management of pest invasions. They can also contribute to bioeconomic analyses that underpin the allocation of resources to alleviate poverty. Because species can invade and establish in areas with climates that are different from those that are found in their native range, it is essential to define robust range-limiting mechanisms in niche models. To avoid spurious results when applied to novel climates, it is necessary to employ cross-validation techniques spanning different knowledge domains (e.g., distribution data, experimental results, phenological observations). We build upon and update a CLIMEX niche model by Parsa et al. (PloS ONE 7: e47675), correcting inconsistent parameters and re-fitting it based on a careful examination of geographical distribution data and relevant literature. Further, we consider the role of irrigation, the known distribution of cassava production and a targeted review of satellite imagery to refine, validate and interpret our model and results. In so doing, we bring new insights into the potential spread of this invasive insect, enabling us to identify potential bio-security threats and biological control opportunities. The fit of the revised model is improved, particularly in relation to the wet and dry limits to establishment, and the parameter values are biologically plausible and accord with published scientific literature.

## Introduction

Cassava (*Manihot esculenta*) is a food staple for millions of people worldwide, and is especially important in Africa, which accounts for half of the global production [1]. Cassava is a primary staple or co-staple food source in much of Africa, with just under 39% of the continent’s consumed food energy deriving from cassava [2]. Cassava production in Africa is increasing in response to famine, hunger and drought, and because of its general resistance to pests and diseases [3]. Furthermore, cassava can be grown on poor soils, is easily propagated and relatively inexpensive to produce [2].

Cassava was introduced to Africa in the 16^th^ century [2]. Until relatively recently, it remained largely free from pests, possibly because it is an introduced species, and possibly because it possesses high quantities of cyanogenic glucosides and latex [4]. The accidental introductions of two cassava pests from South America (the cassava mealybug, *Phenacoccus manihoti*, and the cassava green mite, *Mononychellus tanajoa*) have resulted in serious losses in cassava production as neither of these pests faced the competition and parasitoids of their native range. Both pest species spread rapidly throughout the cassava belt in Africa, causing much more damage than in their native ranges. More recently, the impacts of these insects in sub-Saharan Africa have been compounded by pandemics of begamoviruses (Geminiviridae) vectored by whiteflies in the *Bemisia tabaci* complex [5, 6]. These emerging impacts come at a time when there is growing concern over food security, especially for the poor.

Although *P*. *manihoti* originated in South America and is endemic to the Paraguay River basin, it is patchily distributed, generally occurs in low numbers, and is of no economic importance in this region [7]. Populations of *P*. *manihoti* were not found in Paraguay until 1981 [4, 8] when attempts were made to identify parasitoids that could be used as biological control agents in the African cassava belt, where *P*. *manihoti* was causing severe losses [8]. Although *P*. *manihoti* was thought to occur in Belém, on the northeastern coast of Brazil [9], it would seem that this was a mis-identification, and that the species occurring there is *Phenacoccus herreni* [4, 10].

Populations of *P*. *manihoti* in South America are controlled effectively by a range of parasitoids, and possibly also by competition from other species. This is also apparently the case for *P*. *herreni*, which is distributed throughout South America, but has only caused serious crop losses in northeast Brazil [8, 10]: elsewhere in Brazil it is not a pest [10]. *Apoanagyrus (Epidinocarsis) lopezi* is one of the very effective parasitoids of *P*. *manihoti*, significantly reducing cassava production losses attributable to *P*. *manihoti* in both South America [10] and Africa [4, 11, 12]. Löhr et al. [10] suggest that if *P*. *manihoti* were to be transported to new areas of South America where natural pests do not occur, it would become a major problem. Thus it would seem that parasitoids and competition from other cassava pests both contribute to restricting the populations of *P*. *manihoti* and to preventing it from becoming a significant pest in its native South America.

Knowledge of the potential distribution and relative climate suitability for *P*. *manihoti* could have important implications for the allocation of scarce funding for food security research targeted at improving the livelihood of the world’s poor. Since at least the early 1990s, computer-based models have been popular as a means of estimating the potential distribution of weeds, pests and diseases [13, 14]. CLIMEX has been used to estimate the potential range of a huge number of pests and pathogens, providing reliable prognoses of their future expansion and indicating the regions at risk [15–20]. Parameters can be inferred by fitting stress functions to accord with distribution data, or they can be derived from available experimental observations of laboratory or field data or theoretical principles [21]. Conflicts between information derived from different knowledge domains can be investigated and resolved using the method of multiple competing hypotheses [22]. CLIMEX calculates an Ecoclimatic Index (EI) that describes the overall suitability of locations for population growth and survival. The EI is defined by variables that reflect conditions during the growing season (Growth Index) combined with variables that describe the effects of stresses accumulated during inclement seasons (Stress Index).

We are aware of two previous attempts to model the potential distribution of *P*. *manihoti*. Bellotti et al. [11] developed a bespoke Bioclim-style model based on published experimental observations of *P*. *manihoti* responses to temperature. In the same year, Parsa et al. [23] published a niche model using CLIMEX. The Bellotti et al. [11] model was not described in sufficient detail to allow us to gauge its reliability and suitability for use in bio-economic analyses of the pest threat posed by *P*. *manihoti*. We therefore focused our attention on examining the niche model of Parsa et al. [23], to assess its suitability, and identified issues that rendered it unreliable for the analyses we wished to perform. This model was ostensibly constructed “…to support decision-making in the management of this pest.” [23]. However, we discovered that some of the parameter values used in that model violate both biological principals and accepted methodological practice in the construction of CLIMEX models [21, 24–26]. Furthermore, the model does not correctly project all known locations of *P*. *manihoti* to be suitable, calling into question its suitability to inform pest management decision-making. As with Kriticos et al. [25], updating a previously published CLIMEX model for Siam weed (*Chromolaena odorata*; McFadyen and Skarratt 1996), we have updated the parameter values of Parsa et al. [23] for similar reasons:

1. Because of incorrect model formulation, the modelled climatic suitability is unreliable, and may incorrectly influence management policies.
2. As a published example of a CLIMEX model, it may encourage the continued development of models that are unreliable, and do not reflect good modelling practices.
3. The use of the model as a basis for climate change or similar secondary studies would produce unreliable results.

In re-fitting the CLIMEX model we consider the role of irrigation explicitly, thereby avoiding the distortion of parameters and the simulated phenology of *P*. *manihoti* that is apparent in the original model [23].

## Materials & methods

### Location records

*Phenococcus manihoti* location records were compiled from three sources, and geo-coded for model fitting, verification and validation [27]. For South America, we generated a polygon shape file from Fig 1 in Löhr et al. [10]. For Africa, we generated a point location shape file, using the release sites of *Apoanagyrus (Epidinocarsis) lopezi*, the dominant parasitoid of *P*. *manihoti* in Africa [28], as it seems from Neuenschwander [28] that these releases were all made in areas of high *P*. *manihoti* infestations. For Asia, we used the geo-referenced location records provided by Parsa et al. [23] and Sartiami et al. [29].

**Fig 1:**
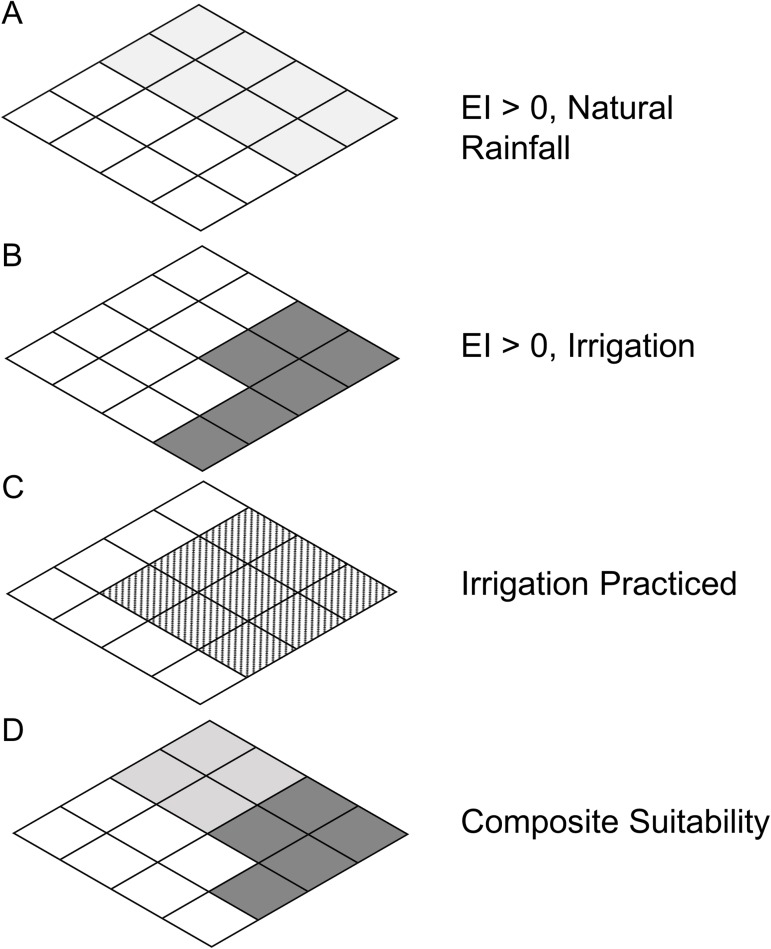
Mapping composite climate suitability. (A) Shaded areas are suitable under a natural rainfall scenario; (B) shaded areas are suitable under an irrigation scenario; (C) hatched areas are the irrigation areas identified by Siebert et al. (2005); (D) composite climate suitability is calculated as the maximum EI values of shaded areas in (A), and shaded areas in (B) where irrigation is practiced [hatched areas in (C)].

### Climate data

For all model runs, we used the CM10_1975H CliMond climatic dataset [30]. This dataset comprises 30-year averages of monthly values for daily minimum and maximum temperature (°C), relative humidity (%) at 09:00 and 15:00, and monthly rainfall total (mm). Apart from being suitable for use with CLIMEX, this also enabled us to directly compare our model with that of Parsa et al. [23], which used the same meteorological dataset.

### Model fitting and validation

We started with the parameter values (Table 1) published by Parsa et al. [23], and adjusted these, initially to fit distribution data for South America [10], as did Parsa et al. [23]. As the Parsa et al. [23] model did not have any Dry Stress (DS) parameters, nor any limiting Heat Stress (HS) in South America, the parameter values for these two stresses were fitted for Africa, using the distribution map of Herren and Neuenschwander [4] and the geo-coded locations from Neuenschwander et al. [28]. Wet Stress (WS) parameters were adjusted so that WS would not accumulate within the parameter bounds suitable for growth, and so as not to be limiting within the known distribution in South America [10] and in Africa [4, 12, 31]. The final model was validated against the distribution points provided for Asia by Parsa et al. [23], Plantwise (http://www.plantwise.org/KnowledgeBank/PWMap.aspx?speciesID=33045&dsID=40173&loc=global), and the recent publication of Sartiami et al. [29]. This validation data was reserved from model fitting, and is geographically independent from the data used to fit the model.

**Table 1:** CLIMEX Parameter Values for *Phenacoccus manihoti*. Values that differ from those of Parsa et al. [23] are given in bold.

Parameter	Description	Parsa et al. 2012 Values	Current Values
**Moisture**			
SM0	lower soil moisture threshold	0	**0.09**
SM1	lower optimum soil moisture	0.01	**0.15**
SM2	upper optimum soil moisture	0.5	0.5
SM3	upper soil moisture threshold	2.5	**2**
**Temperature**			
DV0	lower threshold	16°C	16°C
DV1	lower optimum temperature	24°C	**26°C**
DV2	upper optimum temperature	29°C	**30°C**
DV3	upper threshold	34°C	**35°C**
**Cold Stress**			
TTCS	cold stress temperature threshold		**2.5°C**
THCS	temperature threshold stress accumulation rate		**-0.01**
DTCS	degree-day cold stress threshold	21°C-days	21°C-days
DHCS	degree-day cold stress accumulation rate	-0.0015 week^-1^	**-0.001 week**^**-1**^
**Heat Stress**			
TTHS	heat stress temperature threshold	35°C	
THHS	temperature threshold stress accumulation rate	0.001week^-1^	
DTHS	degree-day heat stress threshold		**5°C-days**
DHHS	degree-day heat stress accumulation rate		**0.003 week**^**-1**^
**Dry Stress**			
SMDS	soil moisture dry stress threshold		**0.09**
HDS	stress accumulation rate		**-0.01week**^**-1**^
**Wet Stress**			
SMWS	soil moisture wet stress threshold	0.8	**2**
HWS	stress accumulation rate	0.00125 week^-1^	**0.009 week**^**-1**^
**Threshold Heat Sum**			
PDD	number of degree-days above DV0 needed to complete one generation	290°C-days	**350°C -days**
**Irrigation Scenario**	2.5 mm day^-1^ as top-up throughout the year		

### Analysis and mapping of results

We adjusted parameter values by only considering a natural rainfall scenario, and used these results to compare our model with that of Parsa et al. [23]. When biologically plausible parameters provided results that accorded with the known distribution, we then ran the model with an irrigation scenario (2.5 mm day^-1^ applied as top-up throughout the year), to capture the risk of attack by *P*. *manihoti* in areas where agriculture is sustained by irrigation practices (i.e., in drier regions of the world). We produced a composite climate suitability map by combining the natural rainfall and irrigation scenario results using the data from Siebert et al. [32] which describe the geographical distribution of irrigation practices. For each 10’ cell, if the irrigated area was greater than 0, the irrigation scenario result was used. Otherwise, the natural rainfall scenario result was used (Fig 1). Removing Dry Stress restrictions and increasing the Soil Moisture Index generally enhances the suitability of drier areas within the projected range, and extends the suitable range into areas otherwise considered to be too dry (i.e., the irrigation area around Nile River will show up as climatically suitable under this irrigation scenario).

To examine our results more closely in regions where cassava is grown, we masked our composite climate suitability map using a spatial union of the total harvested area of cassava (total harvested area > 0) from the two versions of MapSPAM (Spatial Production Allocation Model) [33, 34]. This provides a climate suitability map for all areas where cassava has been known to grow. Our rationale for this choice is that our distribution data for *P*. *manihoti* was collected prior to, and spanning the periods during which the two MapSpam datasets were collated. Therefore, the two datasets combined provided the best estimate of where cassava could have been grown throughout the period in which our *P*. *manihoti* records were collected.

### Parameter adjustment

#### Moisture index

As a plant pest, *P*. *manihoti* cannot be expected to grow under conditions so xeric that growth of its plant host would be impossible. We increased the lower soil moisture threshold for population growth (SM0) to just below the permanent wilting point of plants, which is approximately 0.1 using the CLIMEX 100 mm bucket soil moisture model [35]. This accords with evidence that dry weather, soil moisture stress and soil erosion enhance the population build-up of *P*. *manihoti* [36]; that there are greater infestations on non-mulched plants than on mulched plants [37]; and that all stages do marginally better on stressed plants [4]. Using the same rationale, the lower optimal soil moisture (SM1) was set marginally higher than plant permanent wilting point. The value of the upper optimal soil moisture threshold (SM2) was left unchanged at 0.5: as the upper optimum is likely to be below soil saturation. Similarly, the upper soil moisture threshold (SM3) was reduced to 2, which still allows for persistence in the very wet conditions of Freetown, Sierra Leone [31].

#### Temperature index

The lower development temperature threshold (DV0) was left unchanged at 16°C. The lower optimum threshold temperature (DV1) for population growth was increased to 26°C, based on the data analysis presented in Fig 4 in Schulthess et al. [38]. Similarly, a review of the literature suggested that the upper optimum threshold temperature (DV2) should be increased to 30°C [38, 39]. As Fabres and Boussiengue [40] reported full development of eggs with excellent survival at 34°C, this is unlikely to be the upper threshold, and so we increased the upper threshold for population growth (DV3) to 35°C.

#### Cold stress

The Cold Stress (CS) parameters of Parsa et al. [23] preclude persistence in two locations in Africa (Mbeya in Tanzania, CS = 687 and Serenje in Zambia, CS = 121) [28]. To investigate these anomalies we apply the method of multiple competing hypotheses: “The effort is to bring up into view every rational explanation of new phenomena, and to develop every tenable hypothesis respecting their cause and history.” [22]. Decreasing the rate of stress accumulation (DHCS) to -0.001 makes Serenje suitable, with a non-lethal CS value of 81; however, the CS value of 452 at Mbeya remains lethal. This was considered acceptable for four reasons. Firstly, investigations with Google Earth indicate that agriculture in the area around Mbeya (altitude 1 700 m) occurs in pockets of land at lower altitudes (around 1 600 m). Secondly, as the releases of *A*. *lopezi* [28] were unlikely to have been made in the centre of Mbeya, it is likely that this location does not accurately represent cropping of cassava or the occurrence of *P*. *manihoti* populations. Thirdly, as half of the grid cells adjacent to Mbeya are climatically suitable for the persistence of *P*. *manihoti*, dispersal from these areas could easily result in seasonal populations in Mbeya. Finally, as Serenje and the surrounding agricultural areas have a lower altitude (about 1 400 m), they should be considered suitable for *P*. *manihoti*.

We added a temperature threshold mechanism to the model to simulate the effects of more than one frost per week [25]. This has no impact within the known range of *P*. *manihoti* in either South America or Africa as the frost-sensitive areas on these two continents are almost entirely encompassed by the areas in which CS accumulates via the current degree-day mechanism. Nonetheless, this is a biologically reasonable mechanism to use, as Löhr et al. [10] indicated that populations “suffer serious disruption…from low temperatures”.

#### Heat stress

With a temperature threshold mechanism of Heat Stress (HS), one would generally expect the rate of stress accumulation to be relatively high: i.e., if a temperature *per se* were detrimental, it would result in a negative impact relatively quickly. There is evidence that temperatures above 35°C are detrimental to *P*. *manihoti* [4, 7, 38, 39]. With the upper threshold for development (DV3) now set at 35°C (see above), a HS temperature threshold would have to be at least 35°C since HS cannot accumulate within the range suitable for growth [21, 24–26]. The Parsa et al. [23] parameters (TTHS = 35°C and THHS = 0.001) yield excessive HS estimates along the coast of Guinea and in the region encompassing the borders of Togo, Benin and Burkina Faso, which are infested with *P*. *manihoti* [4, 12]. Different temperature thresholds and rates were considered, and ultimately rejected, because the temperature threshold needed to be increased to 38°C with an accumulation rate of 0.01 so as not to be too restrictive in Africa. The evidence that development and survival is sharply curtailed at 35°C [4, 7, 38, 39] runs counter to any argument that HS would only begin to accumulate and impact the species at 38°C. By contrast, the degree-day HS mechanism (DTHS and DHHS in Table 1) is restrictive along the northern edge of the cassava belt without precluding persistence in any known suitable area. This mechanism enables HS to accumulate when the threshold number of degree-days above the developmental threshold (DV3) of 35°C is exceeded, maintaining a consistency in the temperature at which development ceases and stress impacts on survival.

#### Dry stress

As the lower limiting soil moisture for population growth (SM0) was set at 0 by Parsa et al. [23] they did not use a Dry Stress (DS) mechanism to limit population growth. We set the soil moisture threshold (SMDS) for DS at the same value as SM0 in our model, marginally below the permanent wilting point of plants, since this insect can tolerate conditions stressful to plants [4, 36, 37]. The rate of stress accumulation (HDS) is relatively high, since plants nonetheless need to be alive in order to support populations of *P*. *manihoti*. The reports of *P*. *manihoti* thriving on drought stressed plants are likely to represent plants drawing on stored tuber reserves when soil moisture levels drop below permanent wilting point. There is of course a limit to how long this can be sustained, and there must be a precursor period during which plants have access to adequate soil moisture to create the tuber stores of water and photosynthate. The resulting parameters are therefore a compromise that appears to capture the main features of the ecological system in a manner that accords with the known distribution of *P*. *manihoti*.

#### Wet stress

We adjusted the Wet Stress (WS) parameter values to remove an internal inconsistency in the Parsa et al. [23] model, where WS accumulates within the soil moisture range suitable for population growth. It is no longer acceptable practice in CLIMEX modelling to have stress accumulation occurring within the bounds set for population growth [21, 24–26]. This relationship between growth and stress parameters has been enforced within CLIMEX since version 3. It is only possible to over-ride this default set of relationships for backwards compatibility with older models. The threshold (SMWS) was set to the upper threshold for growth (SM3), and the rate (HWS) was adjusted to allow persistence in Freetown, Sierra Leone [31]. Given the high rainfall in this area here (in excess of 3 000 mm year^-1^), it seems reasonable to allow a high level of WS to accumulate, so long as it does not preclude the persistence of *P*. *manihoti*.

#### Number of degree-days for a feneration (PDD)

We increased the value of PDD to obtain the correct number of generations (9) recorded in Brazzaville [40–42]. The value of 290 degree-days above 16°C used by Parsa et al. [23] allowed 11–12 generations to be completed in this region.

## Results

**Fig 2:**
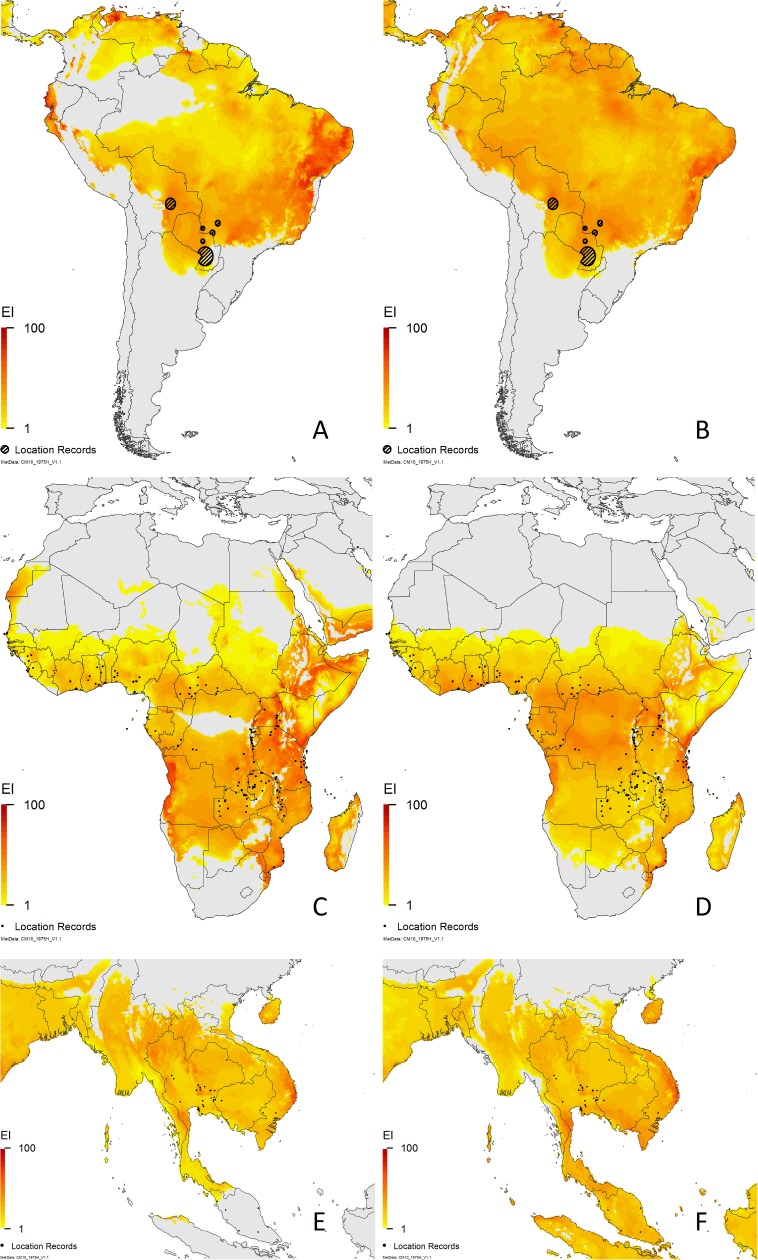
Modelled climate suitability for *Phenacoccus manihoti* under natural rainfall conditions. South America (A, B), Africa (C, D) and south-east Asia (E, F). Maps on the left (A,C and E) were derived using the Parsa et al. [23] parameters; maps on the right (B, D and F) were derived using the new parameter values given in Table 1. Location records were geo-coded from Löhr [10], Neuenschwander [28], Parsa et al. [23] and Sartiami et al. [29].

The differences in the projections of the two models for South America, Africa and Asia are shown in Fig 2. There are clearly major differences in the modelled climate suitability on all continents. The more restricted potential distributions in northwestern South America, central Africa, and in south-eastern Asia using the Parsa et al. [23] parameters result from modelled WS accumulating at an excessively moderate soil moisture level, well within the bounds designated as suitable for growth. Introducing DS further restricts the potential range in North Africa, the Middle East and India, consequently improving the fit of the model by increasing specificity. Even a moderate amount of DS added to any CS or HS accumulated in these areas causes the total stress accumulation to exceed 100, thus returning EI values of 0.

**Fig 3:**
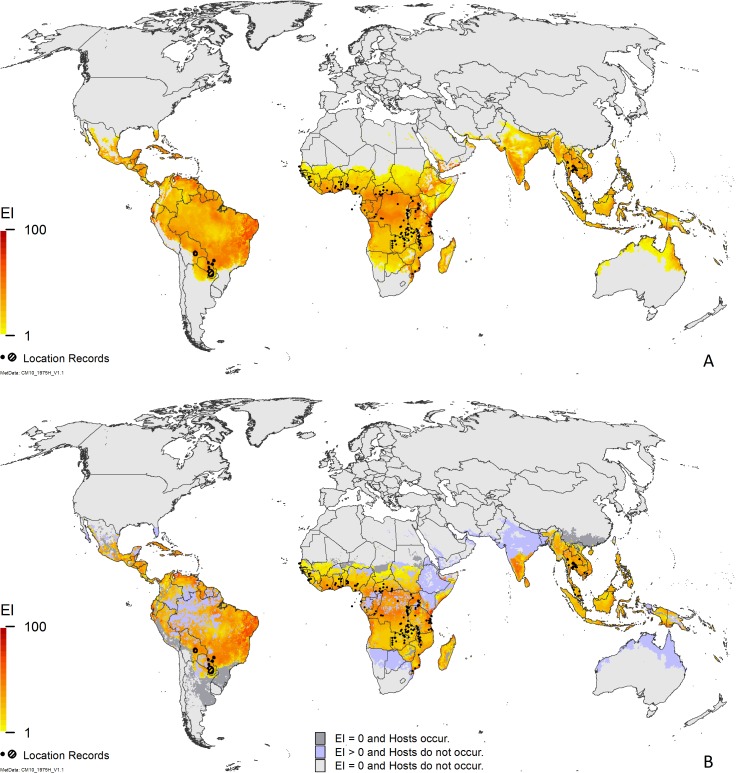
Modelled global climate suitability for *Phenacoccus manihoti*. (A) as a composite of natural rainfall and irrigation based on the irrigation areas identified by Siebert et al. [32]; (B) as a composite of natural rainfall and irrigation based on the irrigation areas identified by Siebert et al. [32] and then masked by harvested areas of cassava [33, 34], highlighting areas of cassava production where *P*. *manihoti* cannot persist and where future expanded cassava production might be at risk from *P*. *manihoti*. Location records were geo-coded from Löhr [10], Neuenschwander [28], Parsa et al. [23] and Sartiami et al. [29].

The composite map, showing the potential global distribution of *P*. *manihoti* is given in Fig 3A, and this same model masked for areas where cassava has been grown is shown in Fig 3B. Fig 3B shows that cassava production occurs in areas that are apparently free from persistent populations of *P*. *manihoti* because these areas are modelled as too cold (Mexico, south and west of South America, central Zimbabwe, Madagascar, and southern China) or too hot (northern Africa). It is interesting to note that in other areas, the suitable climatic range of *P*. *manihoti* exceeds the known extent of cassava cultivation: Fig 3B highlights the areas at potential biosecurity risk from *P*. *manihoti* if cassava production were to be expanded. Whilst most of these are adjacent to areas in which cassava is already grown, Australia stands out in terms of its geographical isolation should cassava production be implemented in the north and west. Central and northern India are also at risk should cassava production be expanded northwards. Although cassava is not widespread as a crop in Ethiopia [43, 44], Namibia [45] and South Africa [46], it is nonetheless grown in these countries, suggesting that the absence of cassava production records in these countries is an error in the MapSPAM dataset. Consequently, cassava production in these countries appears at risk from this pest.

## Discussion

The refined CLIMEX model for *P*. *manihoti* captures the known distribution well, as it encompasses all known location records, including the latest records from Malaysia [29]. Our model displays greater model sensitivity than the model of Parsa et al. (2012), with similar prevalence (specificity), and is created using ecologically plausible parameters that are internally consistent.

Our modelled potential range (Fig 2B) in South America is larger than that modelled by Parsa et al. (Fig 2A), largely owing to a reduction in WS parameters. Although the distribution of *P*. *manihoti* in South America is patchy, and abundance is generally very low, this appears to be the result of high parasitism and predation rates, rather than the influence of climate *per se* [10]. The CS parameters in our model restrict the southwards expansion of *P*. *manihoti* into areas where cassava is harvested. Nonetheless, the area projected to be at risk in South America is greater than the current known extent of *P*. *manihoti*.

It is true that *P*. *manihoti* has not been recorded from the central region of the Democratic Republic of the Congo [47], and Parsa et al. [23] used WS parameters to render that area unsuitable in their model (Fig 2C). However, we dispute that this area is too wet for *P*. *manihoti*. The presence records for *P*. *manihoti* in Malaysia which are rendered unsuitable in the model of Parsa et al. (Fig 2E) occur in climates that are close analogues of the area in the Democratic Republic of the Congo that are also made unsuitable in the model of Parsa et al. (Fig 2C). Refitting the wet stress parameters to remove internal inconsistencies and to accommodate occurrence in Sierra Leone [31] projects suitable climates throughout the Democratic Republic of the Congo as well as Malaysia. Google Earth images show that the central region of the Democratic Republic of the Congo is predominantly forested, with villages appearing to be relatively isolated, although a network of tracks and small roads can be found there. Hennessey et al. [47] suggested that because human settlements in the Congo Basin were few and far between with scarce transport facilities connecting them, the spread of *P*. *manihoti* in this region was limited. This paper also indicated that small, isolated fields in the countryside of the Eastern Shaba region were unaffected by *P*. *manihoti*, whilst cassava fields concentrated around towns had high infestation levels, and that in the southern Shaba Region “…a severe infestation expanded 50–70 km *along the road*…in 1980” [47]. Similarly, Matile-Ferrero [48] remarked that in the Republic of the Congo, areas of infestation followed the railway track linking Brazzaville to Pointe-Noire, and that there were much more limited infestations in isolated areas within the tropical forest. We therefore suggest that climate is not a factor limiting the occurrence of *P*. *manihoti* in the central region of the Democratic Republic of the Congo: rather, dispersal through this region has been severely restricted and the availability and distribution of suitable host plants was exceedingly limited at the time of these surveys.

**Fig 4:**
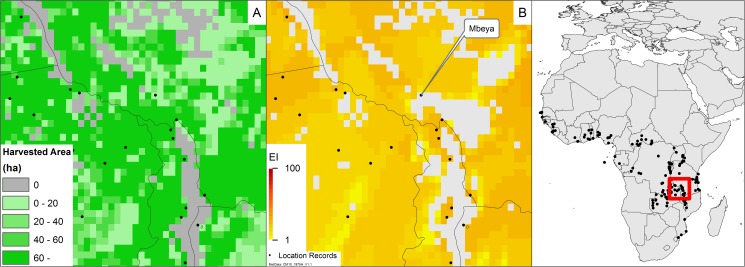
Cassava production and climate suitability for *Phenacoccus manihoti* in southern Tanzania. (A) Average harvested area of cassava in southern Tanzania, produced by a union of the two Map Spam versions [33, 34]. (B) Modelled climate suitability of southern Tanzania for *Phenacoccus manihoti*, as a composite of natural rainfall and irrigation based on the irrigation areas identified in Siebert et al. [32]. Location records are geo-coded from Neuenschwander [28].

We considered the reported harvested areas of cassava production to see if cropping areas match both the location records and the areas indicated as suitable by our model (Fig 3B). The only location record to fall outside the recorded cassava region is Lichinga, in the northwest of Mozambique; however, grid cells surrounding this location that are both climatically suitable and where cassava is grown suggest that this is a spatial precision error within MapSPAM. Of greater interest is the discrepancy found near Mbeya, in southern Tanzania, which is well within the cassava-growing region (Fig 4A), but which is modelled as climatically unsuitable for *P*. *manihoti* (Fig 4B). Areas to the north and west of Mbeya are projected to be climatically suitable for *P*. *manihoti*, whilst areas to the south and east appear climatically unsuitable (Fig 4B) due to excessive CS. We investigated this further, and it would appear that the higher elevation regions to the south and east of Mbeya city are in fact correctly modelled as unsuitable for *P*. *manihoti* (D. Kabungo, pers. comm.).

Modelled excessive CS precludes *P*. *manihoti* from persisting in other areas of Africa where cassava is grown (highland regions of Kenya, Madagascar, Malawi, Tanzania, Zambia and Zimbabwe). In the north of Africa, however, excessive HS precludes persistence of *P*. *manihoti* from areas where cassava is reportedly grown. Strategic sampling or well-designed experiments in areas of the cassava belt indicated by the model as being either too cold or too hot could test the hypothesis that HS and CS are range-limiting in these areas. The results could either validate the model, or provide a basis for re-tuning the CS and HS parameters. Fig 3B indicates that if cassava production were to be extended, a much larger area would be at risk of attack from *P*. *manihoti* (central Africa, large tracts of Ethiopia, Kenya, Somalia, Namibia and Botswana, and the northeastern corner of South Africa).

In Asia, our model performs well, capturing all areas mentioned as having *P*. *manihoti* infestations in Cambodia, Laos and Vietnam [49]. The Parsa et al. [23] model indicates that much of Malaysia, Singapore, Indonesia, Papua New Guinea, and most of the Philippines are unsuitable for *P*. *manihoti* due to excessive WS (Fig 2E), whilst our model indicates that these areas are mostly climatically suitable (Fig 2F). Cassava is produced throughout the lowlands in this region (see Fig 3B), which are climatically similar to equatorial regions in Africa and South America where *P*. *manihoti* occurs. Similar to the situation in the highlands of Africa, cassava is grown in the Maoke Mountain region of Papua, which our model projects to be generally too cold for the persistence of *P*. *manihoti*. Our model indicates that both West Java and Malaysia are climatically suitable (Figs 2F and 3), in accordance with Muniappan et al. ([50], West Java) and Sartiami et al. ([29], Malaysia) recording *P*. *manihoti* from these areas.

Southern China is modelled as unsuitable for permanent populations of *P*. *manihoti*, due to excessive CS accumulation. Systematic surveying in the Chinese provinces in which cassava is grown (Guangxi, Yunnan and Hainan) have confirmed that *P*. *manihoti* was absent [49], and whilst our model suggests that Hainan is suitable, the other areas appear too cold for the establishment and persistence of *P*. *manihoti*. Because this region has a positive GI_A_, introductions of *P*. *manihoti* could result in annual infestations; however, pest management should be possible, with good quarantine practices restricting accidental introductions. It will be interesting to see whether or not *P*. *manihoti* eventually invades China, as the report by Soon and Moekchantuk [49] suggests that both Myanmar and China are of immediate concern as they border countries infested with *P*. *manihoti*.

In summary, the model presented here is internally consistent, with biologically reasonable parameter values. All known location records for *P*. *manihoti* fall within the regions projected to be climatically suitable, with the exception of Mbeya (southern Tanzania). This record is interesting, as it is in a mountainous area where it is apparently too cold to sustain permanent populations of *P*. *manihoti*, but where cassava is grown. With confirmation that this area is indeed not suitable for *P*. *manihoti* (D. Kabungo, pers. comm.), we have to suspect that this record was of an ephemeral (seasonal) population or that it reflects a geo-coding error, as seems likely because the source data [28] only provides the name of the nearest town. CS results in a similar underlap between the pest and host ranges in southern China. In northern Africa, excessive HS restricts the northward distribution of *P*. *manihoti* within the cassava belt. Additional sampling or experimentation in these regions would improve our understanding of the heat and cold tolerances of this species.

To properly fit a niche model, it is necessary to carefully consider the meaning of location records, which frequently contain geo-coding errors, and may also be misleading on a number of other fronts. A location record may arise in a situation that is not representative of the long-term climate averages. For example, irrigation, glasshouses and thermal springs can strongly affect the ability of a population to persist at a location, and as such, distort the relationship between the species presence and the climatic variables being used to drive the climate suitability models. Location records may also reflect observations of an ephemeral population, rather than a permanent one. Modellers need to be ever mindful of these issues, and seek a variety of means of cross-validating records and parameters. We make this point because it is important generally, and because it relates to fitting this particular model. In some cases, while it is possible to fit parameters to accord with distribution data, the parameter values may not be biologically plausible, and it may not be possible to explain the discrepancy. Such situations should be a signal that there is something wrong with the model, and if a plausible explanation cannot be offered, then it is prudent to openly acknowledge such uncertainties. CLIMEX is not simply a mapping utility: it is a powerful set of integrated tools for exploring the mechanisms that are likely to be limiting the distribution of the species being modelled. This study focused considerable attention on questioning the veracity of location records near the modelled range boundaries, and their relationship with fitted model parameters. This approach revealed the anomalies in the model of Parsa et al [23].

*Phenacoccus manihoti* poses a clear and present danger to food production for many of the world’s poorest farmers. It seems clear that when released from the effects of its natural enemies, *P*. *manihoti* is able to expand its range into warmer and wetter areas than within its native range, for example, into areas climatically similar to Brazil. As has been well demonstrated in Africa, this augurs well for efforts to reduce its threat through classical biological control using agents such as *A*. *lopezi* [4, 10–12, 51]. Numerous releases of *A*. *lopezi* in Asia seem to be providing good control of *P*. *manihoti* [29, 52], probably slowing its spread into the adjacent cassava production areas that have not yet been invaded.
